# The Rights of the Majority

**Published:** 1909-11

**Authors:** 


### EDITORIAL DEPARTMENT.

### THE RIGHTS OF THE MAJORITY.

| Misappropriation of Association Funds. | AN OUTRAGE ON THE MEMBERSHIP. | The All Powerful House of Delegates. |
| --- | --- | --- |

### THE TAIL WAGS THE DOG.

Have the members of the State Medical Association any rights that the House of Delegates is bound to respect? Well, yes, if any regard whatever is to be shown the constitution.

The constitution distinctly gives the members—outside of the House of Delegates—the right to participate in the discussion of, and to vote on all questions affecting the organization. Section I, Article VII, says:

"The Association shall hold an annual session during which there *shall be held daily* [italics mine] not less than two general meetings, which shall be open to all registered members," etc. And Article X, providing for a referendum, says:

"The general meeting of the Association may, by a two-thirds vote of the members present, order a general referendum upon any question pending before the House of Delegates; and the House of Delegates may, by a similar vote of its own members, or after a like vote of the general meeting, submit any such question to the membership of the Association for final vote; and if the persons voting shall comprise a majority of all the members [present] a majority of such vote shall determine the question and be binding upon the House of Delegates."

The language is unmistakable. It is mandatory. Yet it has been disregarded, and I do not recall an instance where the majority have been given an opportunity to vote, even on the most vital questions. The yearly volume of Transactions was abolished by the House at a meeting in Houston at which not more than thirty were present, and a Journal established in place thereof. This experiment was tried in 1869—a Journal was published to 1883, inclusive, and the publication of a yearly volume was resumed in 1884. The result is that no one has a record of what was done between 1869 and 1883. There is not a bound volume of the Journals in existence, so far as the committee on records has ever been able to ascertain. Bound volumes of the Transactions from 1884 to 1904—21 years—are available.

The publication of the yearly volume should be resumed ere it is too late, and lest a similar hiatus should occur in the future; for it is believed that not half a dozen members bind the Journals now issued. If they do, it cost them about $4.00 per volume; whereas, the record at the office of the printer in Austin shows, and I have shown more than once, that handsome bound volumes of 650 pages, containing all the papers, discussion, lists of officers, constitution and by-laws, etc., cost only 77 cents and were furnished members, postpaid, free. Resumption of the annual volume—which is the wish of the majority, in my opinion, would not necessarily stop the Journal. It could be issued monthly as a bulletin and have a useful field outside of publishing the papers and discussions. Can not the question be reconsidered at Dallas and the majority be given a voice? The members who prepare papers and travel, in some cases, hundreds of miles to read them, feel sore that their papers do not see daylight in ten or twelve months. Dr. Keiller's excellent paper, read at Corpus Christi in May, 1908, appeared in May, 1909, and another excellent paper read in May, 1908, was published in February, 1909, the author having read the proof in the November previous.

Von Boeckmann-Jones Co. will guarantee to deliver the volumes of Transactions in 90 days.

The House of Delegates at Galveston appropriated (by resolution) $2000 of the members' money—the Association's funds—to enforce the medical practice act! In the name of reason and common sense, what has the Texas State Medical Association—an association of regular physicians—to do with the State Board of (mixed) Medical Examiners? Who made the State Association sponsor for this State law or wet nurse for the Board? This high handed and unwarranted act was without the knowledge or consent of the majority—the men who had contributed the money; and moreover the resolution was adopted over the protest of individual members of the Board of Trustees. *An injunction should be issued to prevent the contemplated misapplication of this trust fund.*

It is needless to say the writer entered his protest at the time, and I quote the official (stenographer's) report of his remarks (*Texas State Journal of Medicine,* June, 1909, page 70):

"Dr. Daniel (said): 'I do not recognize any claim upon the State Association to enforce the State laws. To appropriate a portion of the money which has been collected from the members of the State Association, for such a purpose, would be to levy a direct tax upon every member. This Association has paid in annual dues something like $2000 a year over and above the amount necessary to conduct its business' [here the speaker was interrupted by a point of order]. Resuming, however, he said: 'There is a law against a direct tax, and this membership has been assessed $2.00 per annum for a fund for the Association, and there is a surplus. I can not conceive that it was ever intended that this organization, under our constitution, should divert that fund for any purpose not legal. It is a stretch of the imagination to say that this money can be used for the purpose of enforcing the State law. The law is very emphatic and explicit. It is mandatory. The wording is, that "upon complaint of any member of the Board against any person for unprofessional conduct or conduct calculated to deceive or defraud the public, the court shall prosecute the case, and it shall be the duty of the district attorney or the county attorney to conduct that prosecution."'

Moreover, the law provides that the Attorney General's office shall furnish the Board a lawyer to represent it and the State.

Where, then, does the State Medical Association come in?

Pending the discussion of the resolution to take the members' money to hire a lawyer to enforce a State law, Dr. S. C. Red, one of the Trustees, said that the proposition had been before the Trustees, and that they did "not consider it wise to use the Association's money for this purpose." [*loc. cit.*]

Dr. Cantrell (Trustee) said [*loc. cit.*]: "This is the Trustees' business and we are empowered to attend to it in our own way. We have given the subject mature consideration, and I beg the House to be reasonable in this matter. This old question has been before the Trustees, and in view of all the facts in the case the Board of Trustees did not see their way clear to expend the Association's trust funds in that way. *We have provided, now, more money than the suits in the higher courts require.*" (Italics mine.)

*Nevertheless*—the previous question was moved, seconded, and carried.

Dr. Red fired a final shot thus, but too late: "This House of Delegates would violate the constitution if it passes a resolution of that character." They passed it. As William Lloyd Garrison once said in the Senate: "Constitution be damned."

A discussion of this question took place at Fort Worth, September 3 (1909), at a conference between members of the Examining

Board, the Trustees of the State Medical Association, President Russ, Secretary Chase and Attorney Wilkinson, the Board's counsel, and some others. I make brief extracts from the remarks of Drs. Chase and Red. They are to the effect that the House of Delegates has overridden the constitution and the wishes and opinions of the Trustees.

Secretary-Editor Chase said he "believed the House of Delegates during the debate on this matter at Galveston took action in a moment of enthusiasm without sufficient consideration of the subject," * * * that "the money collected from the doctors of the State, in the treasury of the State Association, is looked upon by the Trustees as a trust fund to advance the interests of the medical profession and is not collected for expenditure in enforcing the laws of the State." [*Texas State Journal of Medicine,* October, 1909, page 241.]

Dr. Red said that the Trustees looked upon the fund in the treasury as a sum to be spent for the elevation of the medical profession —to provide a home for the *Journal,* a library, and other purposes, to advance general professional interests, etc., and that the doctors in his district considered it a *misappropriation of funds* to spend the doctors' money to enforce the State laws." (*loc. cit.*)

Will the majority stand for it?

